# Flexible Indium–Tin Oxide Crystal on Plastic Substrates Supported by Graphene Monolayer

**DOI:** 10.1038/s41598-017-02265-3

**Published:** 2017-06-09

**Authors:** Sang Jin Lee, Yekyung Kim, Jun-Yeon Hwang, Ju-Ho Lee, Seungon Jung, Hyesung Park, Seungmin Cho, Sahn Nahm, Woo Seok Yang, Hyeongkeun Kim, Seung Ho Han

**Affiliations:** 1 Electronic Convergence Materials & Device Research Center, Korea Electronics Technology Institute, Gyeonggi-do, 13509 Korea; 2 Department of Materials Science and Engineering, Korea University, Seoul, 02841 Korea; 3 Carbon Convergence Materials Research Center, Institute of Advanced Composites Materials, Korea Institute of Science and Technology, Jeollabuk-do, 55324 Korea; 4 Reliability Research Center, Korea Electronics Technology Institute, Gyeonggi-do, 13509 Korea; 5 Department of Energy Engineering, School of Energy and Chemical Engineering, Ulsan National Institute of Science and Technology (UNIST), Ulsan, 44919 Korea; 6 New Business Division, Hanhwa Techwin R&D Center, Gyeonggi-do, 13488 Korea

## Abstract

Flexible and crystallized indium–tin oxide (ITO) thin films were successfully obtained on plastic polyethylene terephthalate (PET) films with monolayered graphene as a platform. The highly crystalline ITO (c-ITO) was first fabricated on a rigid substrate of graphene on copper foil and it was subsequently transferred onto a PET substrate by a well-established technique. Despite the plasma damage during ITO deposition, the graphene layer effectively acted as a Cu-diffusion barrier. The c-ITO/graphene/PET electrode with the 60-nm-thick ITO exhibited a reasonable sheet resistance of ~45 Ω sq^−1^ and a transmittance of ~92% at a wavelength of 550 nm. The c-ITO on the monolayered graphene support showed significant enhancement in flexibility compared with the ITO/PET film without graphene because the atomically controlled monolayered graphene acted as a mechanically robust support. The prepared flexible transparent c-ITO/graphene/PET electrode was applied as the anode in a bulk heterojunction polymer solar cell (PSC) to evaluate its performance, which was comparable with that of the commonly used c-ITO/glass electrode. These results represent important progress in the fabrication of flexible transparent electrodes for future optoelectronics applications.

## Introduction

Transparent electrodes are used in various industries, and they are especially popular for application in optoelectronic devices such as flat panel displays, touch sensors, light-emitting diodes (LEDs), and solar cells^1–7^. In the early stage of development of a transparent electrode, a transparent conducting oxide (TCO) is deposited on a rigid substrate like glass. So far, one of the most commonly used materials for TCO is indium–tin oxide (ITO), which exhibits low electrical resistivity and high transparency in the crystalline state. However, amorphous ITO (a-ITO) does not have such properties as those displayed by crystalline ITO (c-ITO). Hence, for application as a TCO, the as-sputtered a-ITO must be transformed to c-ITO by heat treatment at a temperature of at least 250 °C^8, 9^.

Polyethylene terephthalate (PET) is one of the most popular alternatives to rigid glass as a substrate material owing to the increasing demand for flexible, lightweight, and cost-effective transparent electrodes^10, 11^. However, the growth of c-ITO directly on PET is technologically restricted because of PET’s lack of thermal robustness to withstand the high-temperature thermal treatment required for ITO crystallization. Therefore, the preparation of ITO on a PET substrate must be carried out at temperatures below 200 °C, the point at which ITO begins to display poor electrical conductivity and transmittance^12, 13^. An alternative strategy is to synthesize highly crystalline ITO on a rigid substrate at high temperature and then transfer it onto a flexible substrate; successful implementation of this strategy is yet to be reported.

Graphene, a two-dimensional monolayer of sp^2^-bonded carbon atoms, has been intensively researched as a material for flexible transparent electrodes because of its high optical transmittance, electrical mobility, and mechanical flexibility^14^. Although excellent mechanical properties have been reported for graphene^15, 16^, its electrical resistivity is still higher than that of ITO^17^. Therefore, some transparent electrode materials such as ITO^18, 19^ and GaN^20^ have been deposited on graphene transferred onto a PET film, which requires no heat treatment for the electrode fabrication. In the study reported here, we first used graphene on a Cu foil as a rigid substrate for synthesizing highly crystalline ITO. The prepared c-ITO was then transferred onto a flexible substrate. In our study, the graphene layer was utilized as an atomically thin two-dimensional support for obtaining a flexible c-ITO film on a PET substrate. In addition to exhibiting high optical transmittance and mechanical flexibility, the thermal stability of graphene renders it a diffusion barrier of Cu during heat treatment of a-ITO for crystallization. The prepared c-ITO/graphene/PET film was mechanically flexible, while the high optical transmittance and low sheet resistance remained unchanged.

## Results and Discussion

**Figure 1:**
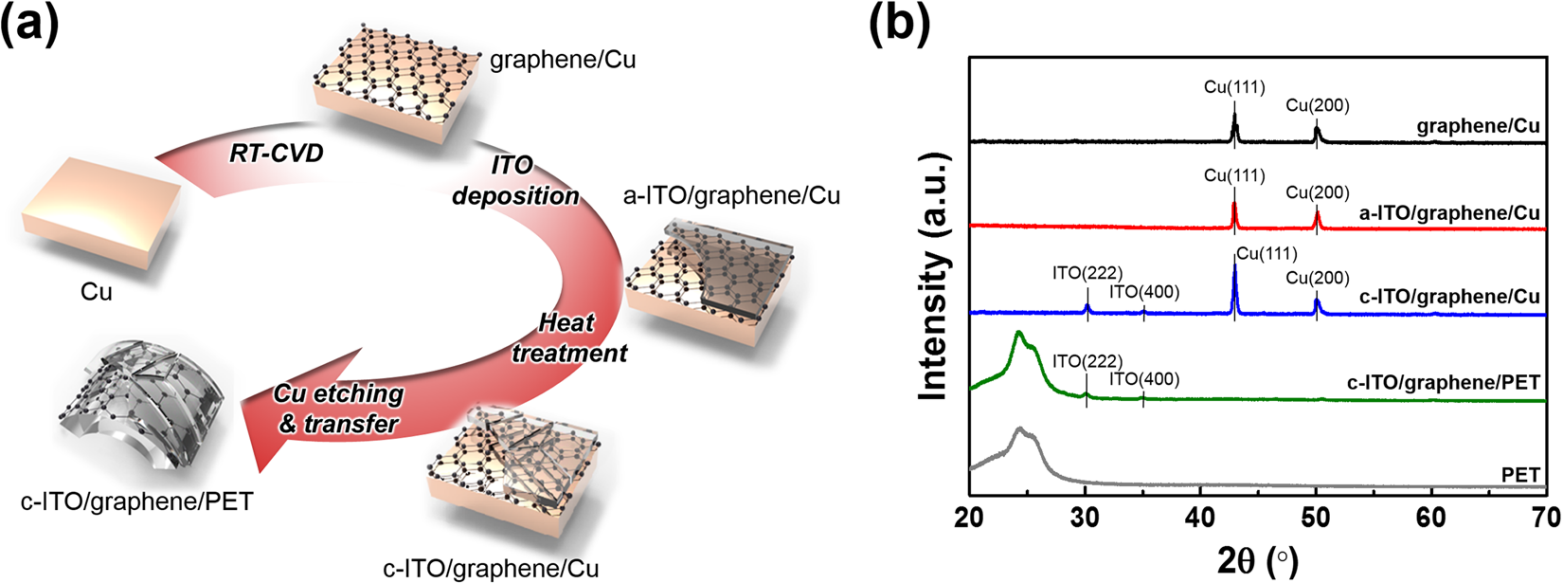
(**a**) Schematic diagram of the fabrication of a c-ITO/graphene/PET film. (**b**) XRD patterns obtained at each step during the preparation of the graphene-supported c-ITO film. The bottom XRD pattern was obtained from the PET film as a reference.

The fabrication procedure for the flexible c-ITO-based films is outlined in Fig. 1a. The flexible c-ITO-based transparent-electrode films were first prepared by the growth of single-layered graphene on Cu foils by rapid thermal chemical vapour deposition (RTCVD)^21, 22^. The a-ITO films were deposited onto the graphene/Cu substrates at various thicknesses of 60, 80, 100, and 120 nm using a direct-current (DC) magnetron sputtering system. Following the a-ITO deposition, the a-ITO/graphene/Cu films were annealed at 250 °C to obtain the crystalline phase. Each graphene-supported c-ITO film was then moved onto a PET film by a well-established graphene-transfer technique^21, 23, 24^.

The crystal structure of each layer was analysed by X-ray diffraction (XRD) (Fig. 1b). The (200) and (111) peaks of Cu were observed in the pattern of the graphene/Cu film. High-quality graphene can be grown with a preferred orientation along the (111) plane of Cu, which has the same hexagonal crystal structure as graphene^25, 26^. The c-ITO formation on the graphene/Cu layer was confirmed by the diffraction peaks at 30.3° and 35.2°, corresponding to the (222) and (400) planes, respectively, of cubic In_2_O_3_ (JCPDS card No. 71-2494), a component of the sputtering target for ITO deposition. As expected, no crystalline ITO peaks were observed in the XRD pattern of a-ITO/graphene/Cu. As shown in Fig. 1b, the peaks of c-ITO also appeared in the XRD pattern of c-ITO/graphene/PET, just as they were observed in the diffraction pattern from c-ITO on graphene/Cu, indicating that the c-ITO/graphene layer was successfully transferred onto the PET substrate.

**Figure 2:**
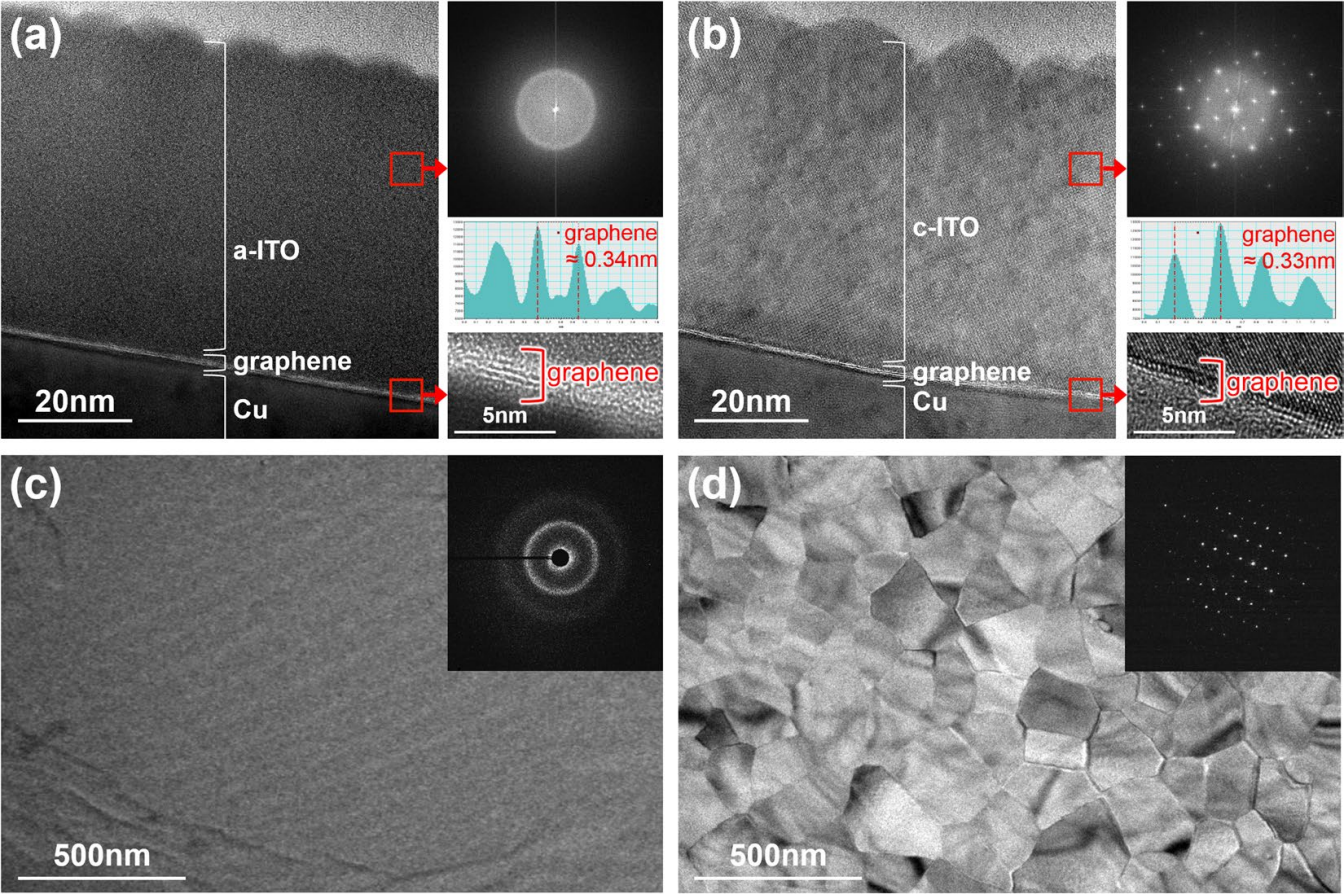
Cross-sectional TEM images of (**a**) a-ITO/graphene/Cu and (**b**) c-ITO/graphene/Cu; corresponding FFT images (upper inset), average intensity profiles (middle inset), and enlarged figures of the graphene layers (lower inset). Plane-view TEM images of (**c**) a-ITO/graphene and (**d**) c-ITO/graphene; the corresponding SADPs are shown in the insets.

The morphology and structure of a-ITO and c-ITO (thickness: ~60 nm) on the graphene support were examined by high-resolution transmission electron microscopy (HRTEM) (Fig. 2). The cross-sectional transmission electron microscope (TEM) images and corresponding fast-Fourier-transform (FFT) patterns of a-ITO and c-ITO on the graphene/Cu substrate confirm their amorphous and crystalline structures, respectively (Fig. 2a,b and upper right insets of Fig. 2a,b). The magnified TEM images and the corresponding average intensity profiles (lower right insets of Fig. 2a and b) clearly show the presence of single to triple graphene layers with an average interlayer distance of ~0.34 and ~0.33 nm, respectively^27^. Figure 2c and d present the plane-view TEM images obtained for a-ITO and c-ITO on graphene, respectively. Details of the specimen preparation procedures for the plane-view TEM images are illustrated in Fig. S1 (see Supplementary information). The steps are summarized as follows: (i) immersing the ITO/graphene/Cu-foil system into an aqueous solution of 0.1 M ammonium persulfate, a Cu etchant; (ii) etching the Cu foil; (iii) mechanically removing the ITO/graphene layer; (iv) mounting the ITO/graphene layer on the TEM grid. As shown in Fig. 2c and d, the low-resolution TEM images and selective area diffraction patterns (SADPs) clearly verify the amorphous structure of the a-ITO layer and crystalline structure of the c-ITO (average grain size: ~200 nm) layer. Based on the HRTEM image of the c-ITO layer, the crystalline phase with (110) and (002) planes at the zone axis of [110] corresponded to the cubic bixbyite structure of the ITO crystal (Fig. S2)^28, 29^.

**Figure 3:**
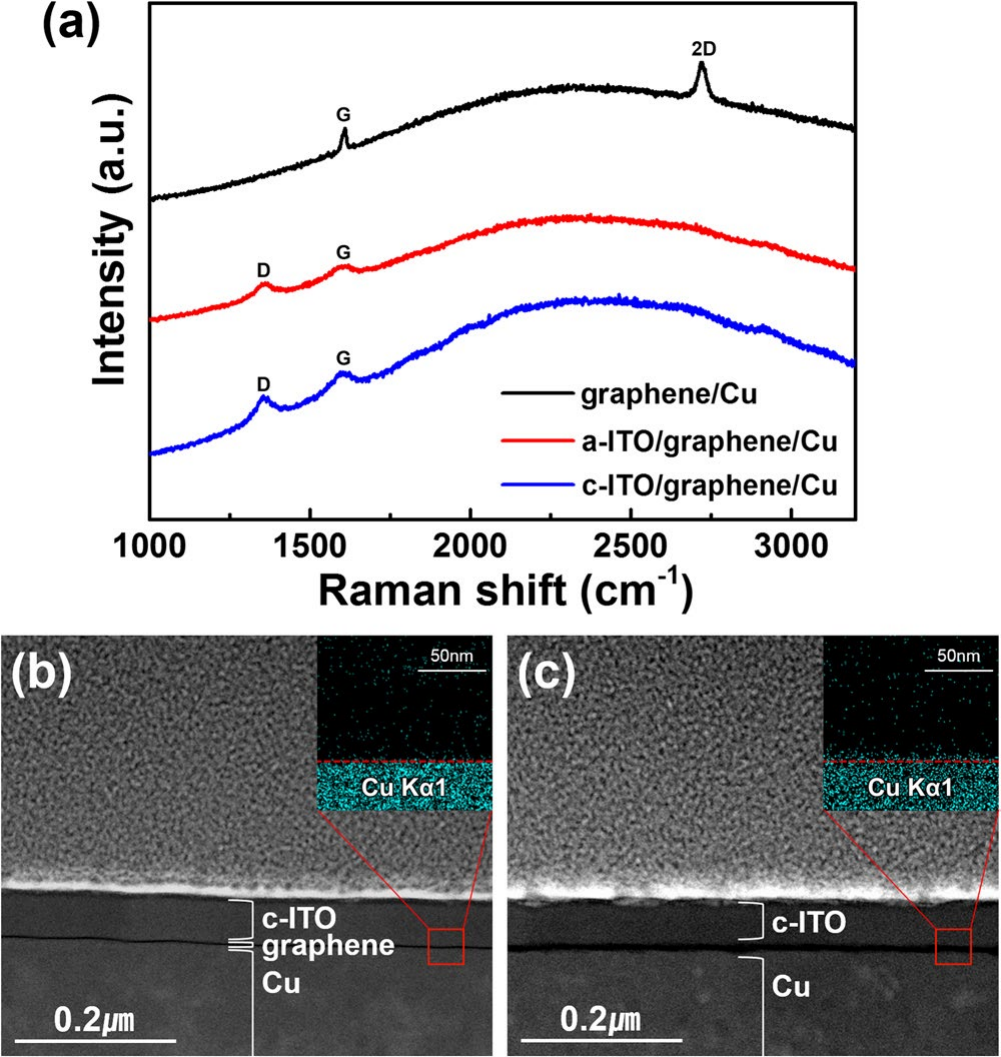
(**a**) Raman spectra of graphene on Cu foil at different states: as-grown, after a-ITO deposition, and after heat treatment for ITO crystallization. Cross-sectional TEM images and EDX elemental mappings of Cu K_α_ for (**b**) c-ITO/graphene/Cu and (**c**) c-ITO/Cu with the c-ITO thickness of 60 nm.

Graphene is known to incur damages as a result of plasma exposure during thin-film deposition^30, 31^. To examine the plasma damage of graphene during ITO sputtering, Raman spectra were obtained for as-grown graphene, graphene after a-ITO deposition, and graphene after crystallization of a-ITO on Cu foil. As shown in Fig. 3a, the G and 2D band peaks appeared at around 1580 and 2680 cm^−1^, respectively, for the as-grown graphene on Cu foil. The symmetric 2D band and minimal D band indicate that the graphene was well-grown, with a negligibly small portion of structural defects, thus qualifying the material for further processing as a flexible transparent electrode^22–24, 32^. However, the 2D peak disappeared and the D peak appeared at 1350 cm^−1^ after a-ITO deposition, which were likely due to the plasma-induced damage in graphene. The ITO crystallization step did not have any effect on the Raman spectra of the a-ITO/graphene/Cu film, suggesting that the structural defects did not propagate and there was no recovery of the graphene. The graphene damage may have induced Cu diffusion into the ITO layer during heat treatment for ITO crystallization. It has been reported that Cu diffusion into the TCO layer results in a decrease in transmittance and conductivity^33, 34^. Figure 3b and c show the TEM images and energy-dispersive X-ray spectroscopy (EDX) mappings of both graphene-supported and graphene-free c-ITO. In contrast to the graphene-supported c-ITO sample (inset of Fig. 3b), the graphene-free c-ITO (inset of Fig. 3c) had a higher Cu content at the bottom interface. This means that despite the damage to graphene by plasma exposure, it still effectively acted as a barrier to Cu inter-diffusion.

**Figure 4:**
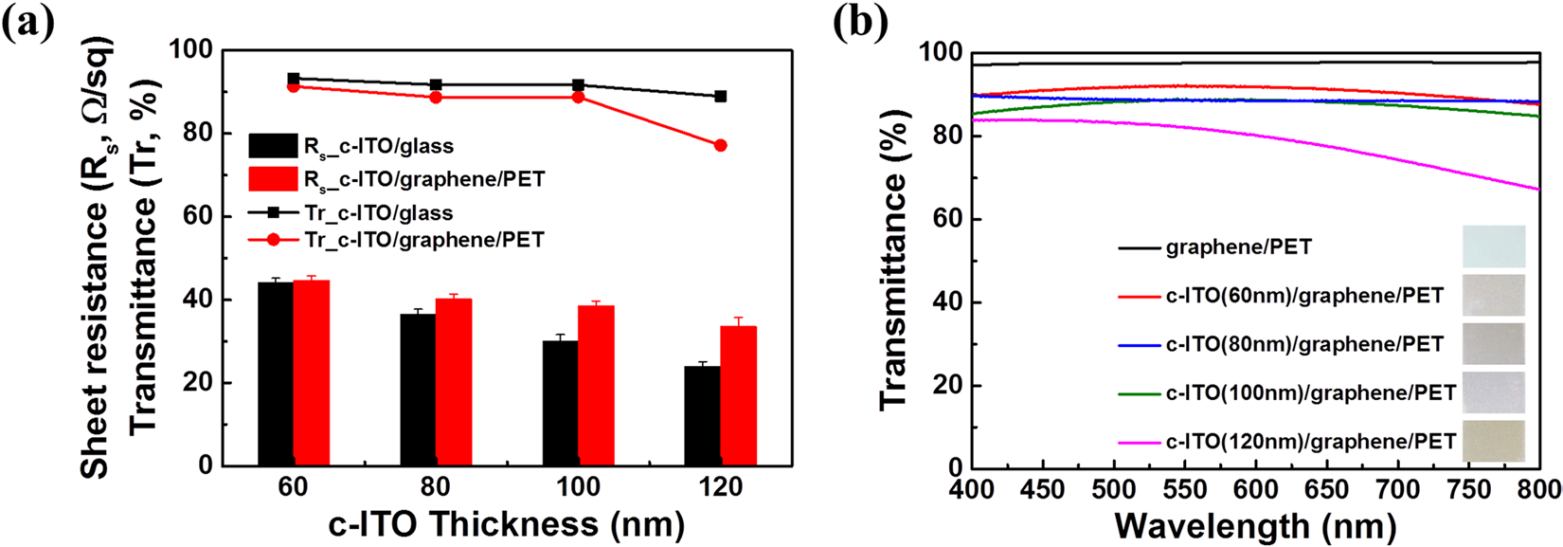
(**a**) Sheet resistance (*R*_s_) and transmittance (Tr; at a wavelength of 550 nm) of the c-ITO on different substrates (graphene/PET and glass). (**b**) Transmittance spectra of the c-ITO/graphene/PET films with various ITO thicknesses; the spectrum of graphene/PET is also shown for comparison.

The electrical and optical properties of the c-ITO/graphene/PET system were evaluated, and the results are shown in Fig. 4. The properties of the c-ITO film on a glass substrate were also measured for comparison. As shown in Fig. 4a, the sheet resistance and transmittance of both c-ITO/graphene/PET and c-ITO/glass decreased with increasing thickness of c-ITO, which are considered to be fairly reasonable results. Figure 4b shows a comparison of the transmittance spectra of graphene/PET and the c-ITO/graphene/PET films with different thicknesses; the specific values of the sheet resistance corresponding to different ITO thicknesses and substrate types are summarized in Table S1. As the thickness of c-ITO increased, the drop in resistance of the c-ITO/graphene/PET film was slower, but its decrease in transmittance was faster when compared to the drops in resistance and transmittance of the c-ITO/glass film. The deteriorating transparent-electrode properties with increasing c-ITO thickness might have resulted from the plasma damage to graphene during ITO deposition. A thicker c-ITO layer corresponded to a longer exposure of graphene to the plasma, which would have resulted in the diffusion of Cu into the c-ITO layer due to the gradual graphene damage. As mentioned above, Cu contamination of ITO generally results in inferior performance of the transparent electrode. Therefore, the sheet resistance and transmittance of the graphene-supported c-ITO with increasing c-ITO thickness were not as high as those of c-ITO on the glass substrate. In the case of the 60 nm-thick c-ITO, the sheet resistances of the c-ITO/graphene/PET and c-ITO/glass systems had similar values of 44.7 and 44.2 Ω sq^−1^, respectively. In addition, the transmittance of c-ITO/graphene/PET (91.3%) was only ~2% smaller than that of c-ITO/glass (93.3%) at a wavelength of 550 nm because of the transmittance and absorbance of visible light by the single-layered graphene^35^. Consequently, the plasma exposure for deposition of up to a 60-nm-thick ITO layer was not long enough to damage the graphene. Nevertheless, the low sheet resistance of 44.7 Ω sq^−1^ and high transmittance of 91.3% have been beyond the reach of other ITO/PET systems^22, 31^.

**Figure 5:**
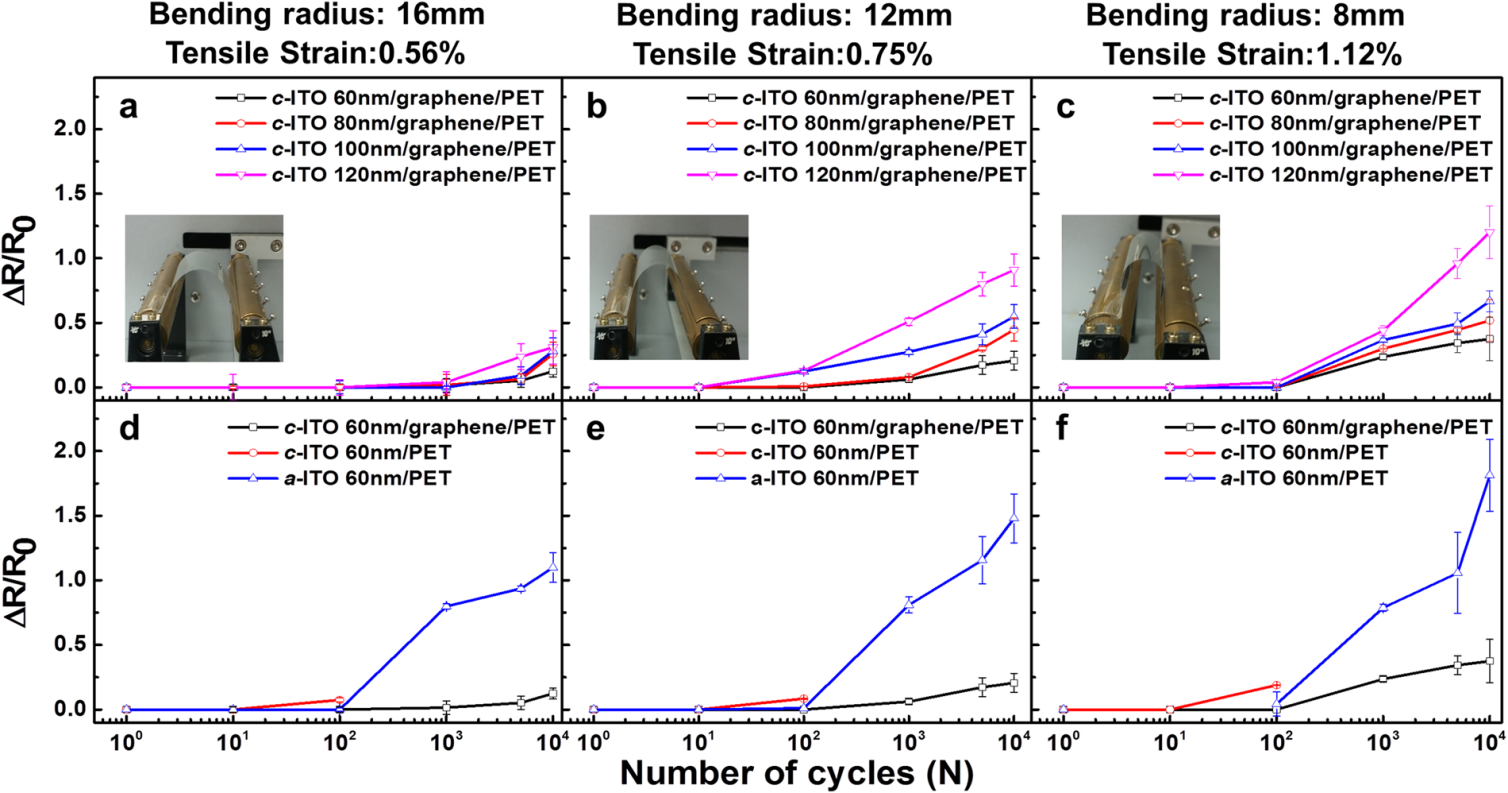
Normalized change in resistance, Δ*R*/*R*_0_, as a function of the number of bending cycles for the c-ITO/graphene/PET films with various ITO thicknesses at bending curvature radii/tensile strains of (**a**) 16 mm/0.56%, (**b**) 12 mm/0.75%, and (**c**) 8 mm/1.12%. Δ*R*/*R*_0_ as a function of the number of bending cycles for c-ITO/graphene/PET, c-ITO/PET, and a-ITO/PET with the same ITO thickness of 60 nm at bending curvature radii/tensile strains of (**d**) 16 mm/0.56%, (**e**) 12 mm/0.75%, and (**f**) 8 mm/1.12%.

The electromechanical durability was evaluated by applying repeated tensile strain to the electrodes composed of graphene-supported c-ITO films with c-ITO thicknesses of 60, 80, 100, and 120 nm. This was accomplished by bending the films to radii of curvature of 16, 12, and 8 mm, thus inducing tensile strains of 0.56, 0.75, and 1.12%, respectively, in the top c-ITO layer. Meanwhile, the normalized resistance change, Δ*R*/*R*_0_ (where *R*_0_ is the initial resistance, and Δ*R* is the difference in the resistances before and after the bending test) was measured as a function of the number of bending cycles (*N*); the results are shown in Fig. 5a,b and c, respectively. No electromechanical instability was observed in any of the samples for the first 10^2^ bending cycles with the highest strain of 1.12% (8 mm bending radius), and thereafter, the resistances changed by less than 5% of their initial resistance. Although Δ*R*/*R*_0_ changed gradually with repeated bending, the film with a 60-nm-thick layer of c-ITO showed the smallest variations. As mentioned earlier, the graphene damage with increasing c-ITO thickness might have been responsible for the gradual increase in Δ*R*/*R*_0_ as the number of bending cycles increased. Because graphene has extremely high strength and stiffness^36^, it effectively acted as a mechanically robust support between the ITO film and PET substrate^18^. In addition, the interfacial C–O bonding between graphene and the oxygen atoms of ITO^37^ induced a strong attachment of the ITO layer to the graphene substrate and prevented fractures in the c-ITO. As a result, the graphene-supported 60-nm-thick c-ITO film had the best electromechanical stability against bending stress owing to the relatively low plasma damage to the graphene layer. Meanwhile, despite the slight increase in the c-ITO film thickness (from 60 to 120 nm), the gradual damage in graphene with increasing c-ITO thickness resulted in a steeper increase in Δ*R*/*R*_0_.

Figure 5d,e and f show the normalized change in resistance of c-ITO/graphene/PET, c-ITO/PET, and a-ITO/PET with the same ITO thickness of 60 nm as a function of the number of bending cycles when the films were subjected to bending radii of 16, 12, and 8 mm, respectively, inducing tensile strains of 0.56, 0.75, and 1.12%, respectively. The graphene-free a-ITO/PET film was fabricated by depositing ITO directly on the PET substrate without any heat treatment. In contrast to the small variations in Δ*R*/*R*_0_ for the first 10^4^ bending cycles of the graphene-supported c-ITO film, the graphene-free c-ITO/PET film cracked after 10^2^ bending cycles for all values of the bending radius. Furthermore, the resistance changes of the graphene-free a-ITO/PET increased remarkably after 10^2^ bending cycles. From the above analysis, it is obvious that the graphene support effectively enhanced the electromechanical stability of the c-ITO film against the bending stress, which is attributed to the robust adhesion of the c-ITO layer on the graphene support.

**Figure 6:**
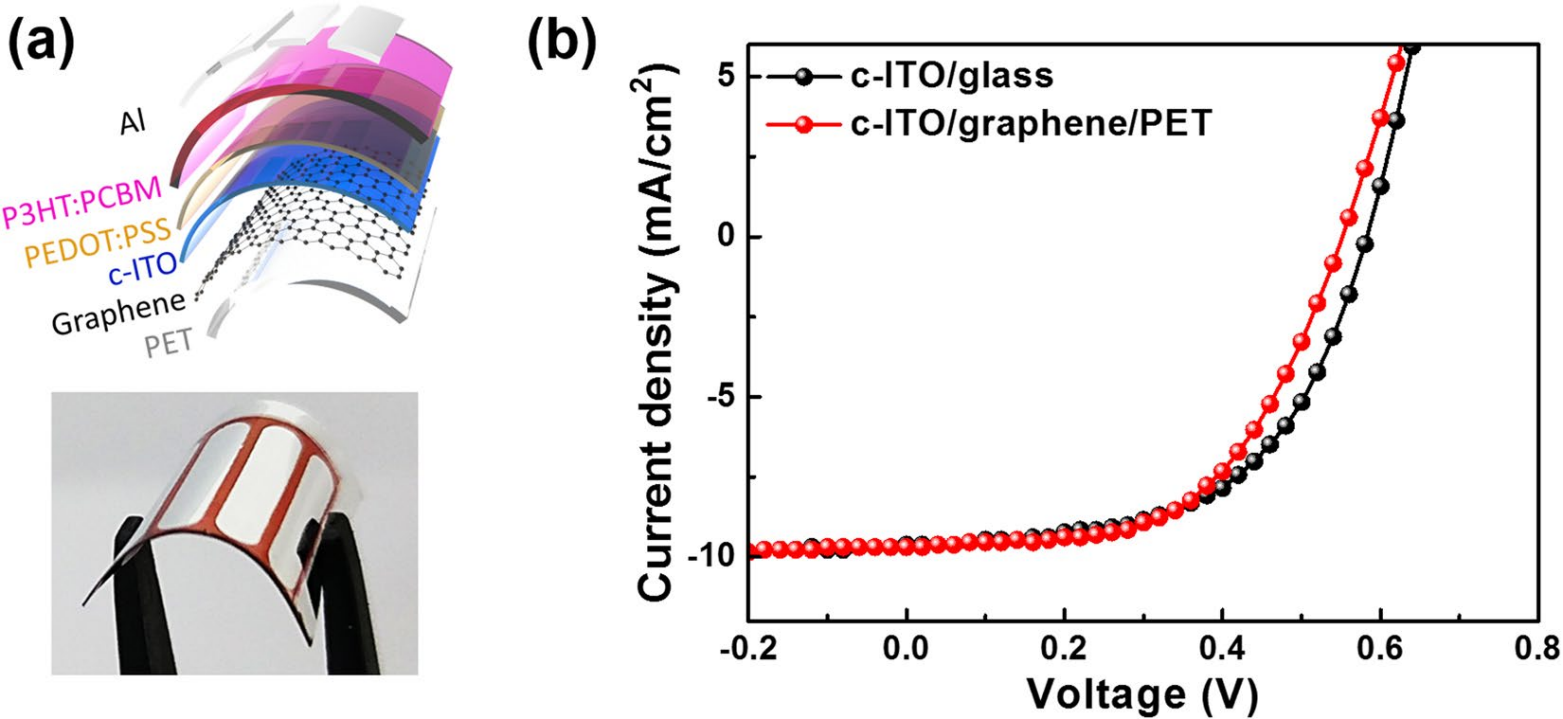
(**a**) Schematic illustration of the device structure and digital image of the PSC prepared with a graphene-based c-ITO anode on a PET substrate. (**b**) Current density–voltage (*J*–*V*) curves of the PSCs with c-ITO/graphene/PET and c-ITO/glass as the anode materials.

The graphene-supported 60-nm-thick c-ITO film was evaluated as a transparent electrode of a bulk heterojunction polymer solar cell (PSC), as illustrated in Fig. 6a. A PSC prepared on a rigid glass substrate with the same thickness (60 nm) was also evaluated as a reference. The hole-transporting medium of poly(3,4-ethylenedioxythiophene)–polystyrene sulfonate (PEDOT–PSS) was spin-coated at 4000 rpm for 60 s on the anode material (i.e. c-ITO/graphene/PET or c-ITO/glass); the PEDOT–PSS layer was then annealed at 110 °C. Next, a photoactive medium consisting of a mixture of poly(3-hexylthiophene) (P3HT) and phenyl-C61-butyric acid methyl ester (PCBM) in o-dichlorobenzene (DCB), was spin-coated onto the PEDOT–PSS layer at 1500 rpm for 60 s, followed by annealing at 110 °C. Finally, an Al cathode with a thickness of 100 nm was deposited by thermal evaporation. The current density (*J*)–voltage (*V*) characteristics of the prepared PSCs are shown in Fig. 6b, and the performance parameters are summarized in Table 1. As shown in Fig. 6, the c-ITO/graphene/PET anode acted as an effective rectifying diode, and the resulting PSC showed photo-response characteristics similar to those of the PSC based on the reference c-ITO/glass anode. The maximum power-conversion efficiencies (PCEs) of the PSCs incorporating the c-ITO/graphene/PET and c-ITO/glass anodes were 3.0% and 3.1%, respectively. The other performance parameters, such as short-circuit current density (*J*_sc_), open-circuit voltage (*V*_oc_), and fill factor (FF), were also very similar. Such comparable performance of c-ITO/glass and c-ITO/graphene/PET as the transparent conducting electrode is advantageous for various flexible device applications because of the c-ITO/graphene/PET system’s superior flexibility and excellent bending stability.

**Table 1:** Performance of PSCs fabricated with c-ITO/graphene/PET and c-ITO/glass as the anode materials using the 60-nm-thick c-ITO film.

Anode substrate	*J*_SC_ (mA cm^−2^)	*V*_OC_ (V)	FF (%)	PCE (%)
c-ITO/glass	9.62	0.58	55.61	3.13
c-ITO/graphene/PET	9.69	0.55	55.37	2.96

In summary, we demonstrated the successful fabrication of a flexible c-ITO electrode with the aid of single-layered graphene support on a PET substrate. The atomically thin graphene film acted as a mechanically robust support for enhanced flexibility, as well as a thermal diffusion barrier against Cu diffusion into the ITO layer. The c-ITO/graphene/PET electrode exhibited sheet resistance and transmittance that were highly comparable with those of the c-ITO/glass electrode; it also showed significantly enhanced bending stability compared to that of the ITO/PET electrode without a graphene support. The fabricated c-ITO/graphene/PET electrode was used in a bulk heterojunction PSC, and the resulting PSC showed device performance comparable to that of the PSC with the c-ITO/glass electrode. These results suggest that c-ITO/graphene/PET is a promising flexible transparent electrode material for a variety of next-generation flexible optoelectronic devices. Also, the electrode transferring technique suggested in this study could bring out a technical issue of transferring an already-prepared module from a copper surface to a flexible or transparent substrate. As a next step for this research, a whole electrical cell could be fabricated on a copper substrate, and then transferred to a transparent substrate by this electrode transferring technique.

## Methods

### Preparation of graphene-supported flexible c-ITO electrode

Single-layered graphene was grown on copper foil (99.8%) by RTCVD (Fig. S3a). The Cu-foil substrate was rapidly heated to around 1030 °C under an Ar flow at 30 sccm and 600 mTorr. Once the temperature reached 1030 °C, the Cu foil was annealed for 1120 s, after which CH_4_ was supplied to the chamber at 30 sccm for 500 s while maintaining the temperature for graphene growth. After the graphene growth, the chamber was cooled to room temperature^21, 22^.

ITO was deposited on the graphene/Cu sample using a DC magnetron sputtering system (T-504, SUKWON Co., Ltd., Korea). A 4-in disk of In_2_O_3_–SnO_2_ (Sn concentration: 10 wt%) was used as the ceramic sputtering target for the ITO deposition. The deposition conditions were as follows: DC power, 500 W; Ar to O_2_ ratio, 30:0.5; working pressure, below 2.5 × 10^−6^ Torr. The film thickness was varied from 60 to 120 nm. The as-deposited a-ITO/graphene/Cu electrode was then annealed at 250 °C to form c-ITO/graphene/Cu (Fig. S3b).

For the transfer of the graphene-supported c-ITO onto the PET substrate, the as-prepared c-ITO/graphene/Cu film was first attached to a piece of thermal release tape. Using a standard wet etching method, the bottom Cu foil was then etched with an aqueous 0.1 M ammonium persulfate solution. After the residual etchant was removed several times with de-ionized water, the remaining graphene-supported c-ITO (c-ITO/graphene) on the thermal release tape was placed on the PET film with the graphene side facing the PET surface. A set of rollers was used to apply pressure on the back side of the thermal release tape and laminate the c-ITO/graphene film on the PET film at a mild temperature of 120 °C. The c-ITO/graphene film was transferred to the PET film as the thermal release tape lost its adhesive force at the elevated temperature. An a-ITO/graphene/Cu film was also prepared using the same procedure, but without the annealing process.

### Characterization

The crystal structure was identified by XRD (Seifert XRD 3000, GE Sensing & Inspection Technologies GmbH, Germany) with Cu K_α_ radiation (*λ* = 1.54056 Å). The cross section and surface morphology of the graphene-supported ITO films were examined with a transmission electron microscope (Titan G2 60–300, FEI, USA) equipped with an energy-dispersive X-ray spectrometer (Super-X EDX, Bruker, USA). The specimens for the cross-sectional TEM analysis were prepared with a focused ion beam. The TEM was operated at 80 kV, and the EDX signal was collected at around 4.0 nA with a convergence angle of 13.5 mrad. The structure of the graphene layer in the specimens was monitored by a Raman spectrometer (inVia Raman microscope, Renishaw, USA) operated at 2.41 eV, with an excitation wavelength of 514 nm. The transmittance of the graphene-supported ITO films was measured with ultraviolet–visible–near-infrared (UV–vis–NIR) spectrophotometer (V-670, JASCO, Japan). The sheet resistance was measured with a four-point probe (FPP-RS9, DASOLENG Co., Ltd., Korea). Using a bending tester (ZRT-200, Z-tec Co., Ltd., Korea), the electromechanical bending test was performed by measuring the change in sheet resistance as a function of the number of bending cycles with various bending radii.

## Electronic supplementary material

Supplementary Information. 
