# Supplementary material for: Flexible Indium–Tin Oxide Crystal on Plastic Substrates Supported by Graphene Monolayer
Source: Sci Rep. 2017 Jun 9;7:3131. doi: 10.1038/s41598-017-02265-3 (PMC5466659; doi:10.1038/s41598-017-02265-3)
Supplement: Supplementary file 1 — Supplementary Information. [file 41598_2017_2265_MOESM1_ESM.doc]

Supplementary Information

Flexible Indium–Tin Oxide Crystal on Plastic Substrates Supported by Graphene Monolayer

Sang Jin Lee, Yekyung Kim, Jun-Yeon Hwang, Ju-Ho Lee, Seungon Jung, Hyesung Park, Seungmin Cho, Sahn Nahm, Woo Seok Yang, Hyeongkeun Kim and Seung Ho Han


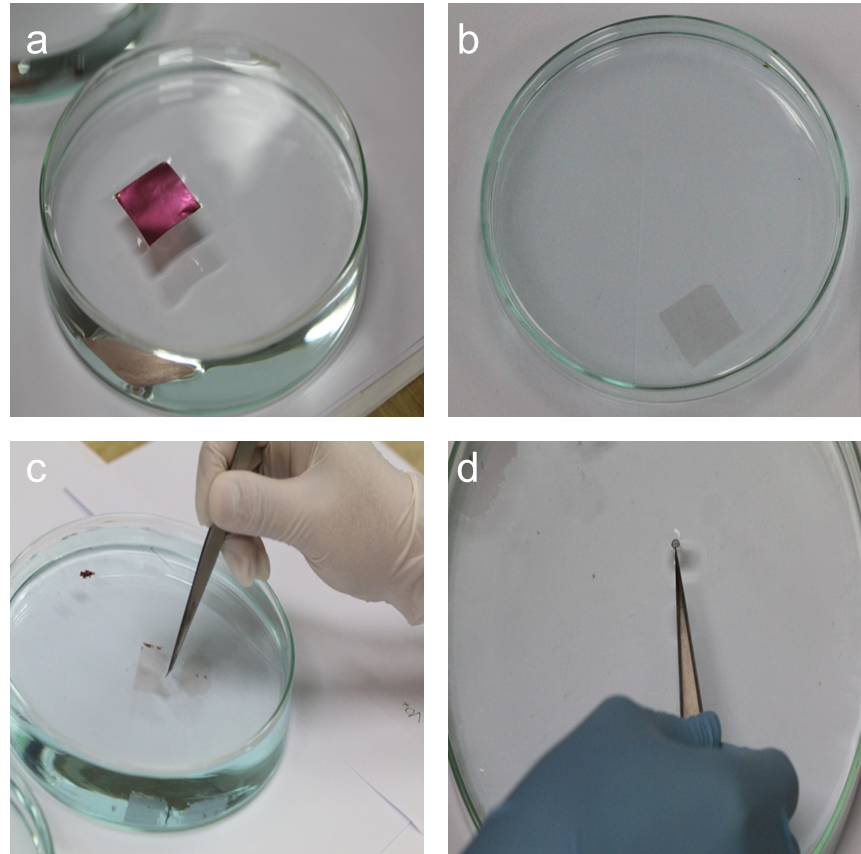


**Supplementary Figure S1.** Preparation for plane-view TEM specimen by transferring ITO/graphene layer from Cu foil into TEM grid; (a) floating ITO/graphene/Cu foil on 0.1 M ammonium persulfate solution, (b) etching out the Cu foil, (c) mechanically tearing down the ITO/graphene, and (d) dipping up on the TEM grid.


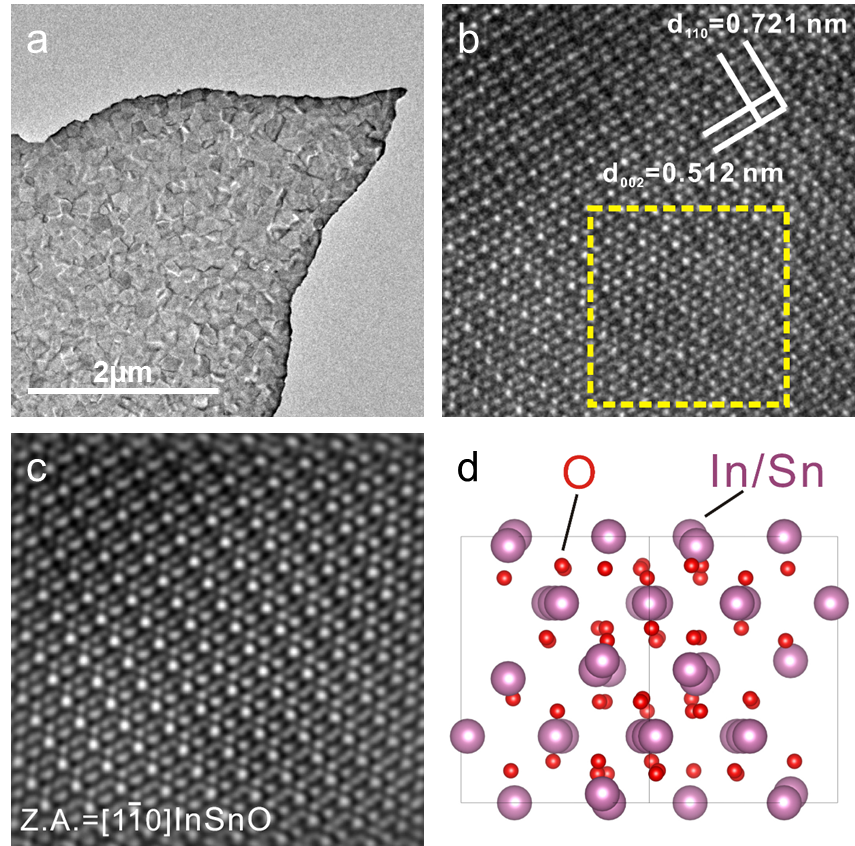


**Supplementary Figure S2** Plane-view TEM images of the c-ITO/graphene film; (a) the low-resolution, (b) high-resolution, and (c) enlarged view of (b). (d) Schematic cubic bixbyite structure of the ITO crystal.


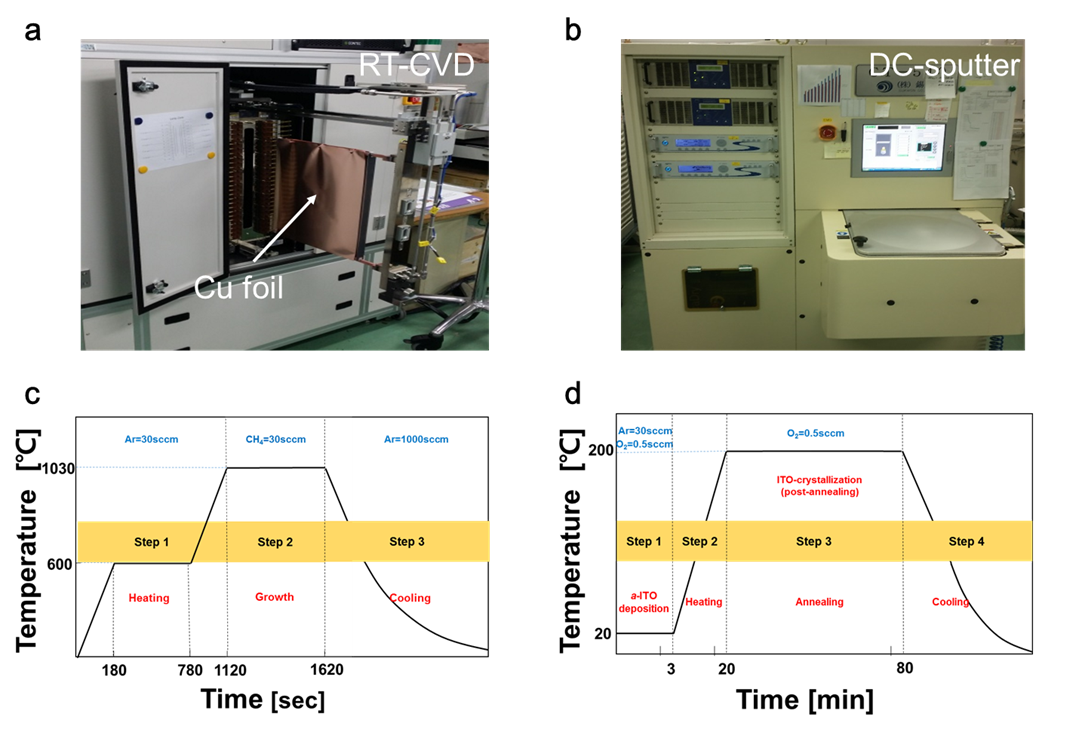


**Supplementary Figure S3** The pictures of (a) RT-CVD system loaded with two sheets of Cu foil (350×480 mm^2^) for the synthesis of mono-layer graphene and (b) DC-magnetron sputtering system (maximum loading size of 6 inch wafer) used for ITO deposition, and the process sequence of (c) graphene growth condition using (a) and (d) ITO deposition using (b).


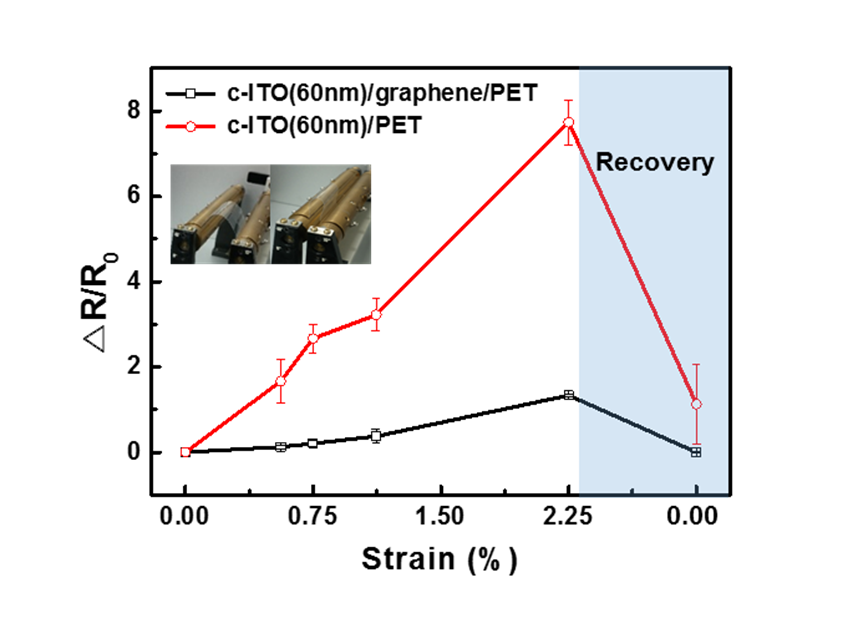


**Figure S4.** Normalized change in the electrical sheet resistance of the electrodes deposited with 60-nm-thick c-ITO, with (black) and without (red) the graphene layer, as a function of the tensile strain, including the effect of strain release (blue background) after applying the highest strain. The sheet resistance of c-ITO/PET drastically increases with increasing tensile strain, and the resistance does not recover after removing the stress. In contrast, the c-ITO/graphene/PET film exhibits more stable sheet resistance, which only increases by 33% at a tensile strain of 2.25%. Moreover, the resistance fully recovers after releasing the strain force. In conclusion, as a buffer layer, the graphene monolayer enhances the electromechanical strength of the electrode.

**Supplementary Table S1** The sheet resistances values of the c-ITO both on a glass and graphene/PET substrates with various c-ITO thicknesses.

|  | **Sheet resistance (Ω/sq)** | | | |
| --- | --- | --- | --- | --- |
|  | **60 nm** | **80 nm** | **100 nm** | **120 nm** |
| **c-ITO/glass** | 44.7 | 40.2 | 30.1 | 24.0 |
| **c-ITO/graphene/PET** | 44.2 | 36.5 | 38.6 | 33.6 |
